# Stereotactic lesioning combined with long-term Risperidone maintenance for life-threatening refractory suicidal command hallucinations in major depressive episode with psychotic features: a case report

**DOI:** 10.1186/s12888-025-07627-0

**Published:** 2025-11-19

**Authors:** Cheng Chen, Ruiting Li, Hanping Bai, Yunlong Peng, Huiling Wang

**Affiliations:** 1 Department of Psychiatry, Renmin Hospital of Wuhan University, Wuhan, 430060 China; 2 Department of Psychiatry, Yidu People’ s Hospital, Yidu, 443300 China; 3 Hubei Institute of Neurology and Psychiatry Research, Wuhan, 430060 China

**Keywords:** Stereotactic lesioning, Suicidal command hallucinations, Major depressive episode with psychotic features, Postoperative pharmacotherapy, Long-term maintenance treatment

## Abstract

**Background:**

Refractory suicidal command hallucinations in major depressive episode with psychotic features constitute an acute clinical emergency, often unresponsive to multiple antipsychotics and electroconvulsive therapy (ECT), leaving clinicians with limited life-saving options. Stereotactic lesioning, as a last-resort intervention, may have some utility in such case; however, its long-term efficacy and the extent to which it depends on postoperative pharmacotherapy are not yet fully understood.

**Case presentation:**

A 20-year-old female with major depressive episode with psychotic features experienced 10–12 daily suicidal command hallucinations (with 4 suicide attempts within 1 week), refractory to 3 antipsychotics (Aripiprazole, Olanzapine, Risperidone) and 12 sessions of ECT (Montgomery-Asberg Depression Rating Scale [MADRS]: 30, Hoffman auditory hallucination score [Hoffman]: 25). Stereotactic bilateral lesioning of the anterior limb of the internal capsule and cingulate gyrus (SALIC-CG) resulted in a marked alleviation of symptoms within 1 week (MADRS: 7, Hoffman: 0). Notably, symptoms recurred 1 month postoperatively following antipsychotic discontinuation (MADRS: 22, Hoffman: 20) but resolved rapidly upon reintroducing Risperidone, with sustained remission at 6 months (MADRS: 6, Hoffman: 0) under continuous pharmacotherapy.

**Conclusions:**

This case offers preliminary evidence that stereotactic lesioning may act as a key intervention for alleviating symptoms in patients with psychotic major depressive episode—specifically those with intractable suicidal command hallucinations that do not respond to all conventional treatments. Critically, it underscores that postoperative long-term antipsychotic maintenance is not merely adjunctive but essential to preserve surgical benefits, establishing a “surgery + long-term pharmacotherapy” paradigm with direct implications for managing high-risk refractory cases.

**Clinical trial number:**

It is not a clinical trial, clinical trial number: not applicable.

## Introduction

Major depressive episode with psychotic features, a severe subtype of major depressive disorder, affects 20–30% of patients with major depression, characterized by concurrent severe depression and psychotic symptoms (e.g., delusions, auditory hallucinations) [1]. Among these symptoms, suicidal command hallucinations are particularly perilous: tightly intertwined with depressive cognitions (e.g., “punishment for worthlessness”), they are associated with a 4.2-fold higher suicide risk than non-psychotic depression [2, 3]. Refractory cases—defined as unresponsiveness to ≥ 2 standard treatments—pose an acute clinical emergency, as conventional interventions (second-generation antipsychotics, mood stabilizers, electroconvulsive therapy [ECT]) fail to mitigate risk in 15–20% of patients [4, 5].

Stereotactic lesioning, targeting dysregulated fronto-limbic circuits (e.g., anterior limb of the internal capsule [ALIC], cingulate gyrus) involved in emotional processing and impulsivity, has emerged as a potential last-resort intervention [6, 7]. However, its efficacy in major depressive episode with psychotic features—particularly in cases involving high-risk refractory suicidal command hallucinations—and the critical role of postoperative pharmacotherapy in sustaining therapeutic benefits remain poorly characterized, leaving significant gaps in clinical evidence.

Here, we report a unique case of life-threatening refractory suicidal command hallucinations in a patient with psychotic major depressive episode, which had proven unresponsive to multiple antipsychotics and electroconvulsive therapy (ECT). This patient achieved remission following stereotactic lesioning combined with long-term risperidone maintenance. By focusing on this high-risk, treatment-refractory presentation, our case validates the “surgery + long-term pharmacotherapy” paradigm and contributes valuable real-world data to address the existing gaps in postoperative pharmacotherapy research for this challenging patient population.

## Case presentation

A 20-year-old female was hospitalized for low mood and auditory hallucinations accompanied by suicidal behaviors. Psychiatric examination revealed depressed mood, command hallucinations, and a diagnosis of major depressive episode with psychotic features according to the International Classification of Diseases-10 (ICD-10).

During the period from 2022 to 2023, she was hospitalized multiple times due to obvious emotional instability and command hallucinations, and the drugs such as Aripiprazole or Olanzapine did not show satisfactory results for her treatment. Partial improvement in hallucinations followed Risperidone initiation, but symptom exacerbation occurred after discontinuation, including recurrent command hallucinations, self-talk, negative thoughts, and frequent self-harm/suicide attempts (Table 1).

**Table 1:** Previous history of Pharmacological interventions and outcomes

Drug Regimen	Efficacy	Adverse Effects	Clinical Action
Aripiprazole 20 mg/day	No therapeutic effect	Tremor, Akathisia,	Regimen discontinuation
Olanzapine 20 mg/day	Poor therapeutic effect	Metabolic syndrome	Self-discontinuation
Risperidone (4 mg/day)	Partial response	Drowsiness	Self-discontinuation

**Fig. 1:**
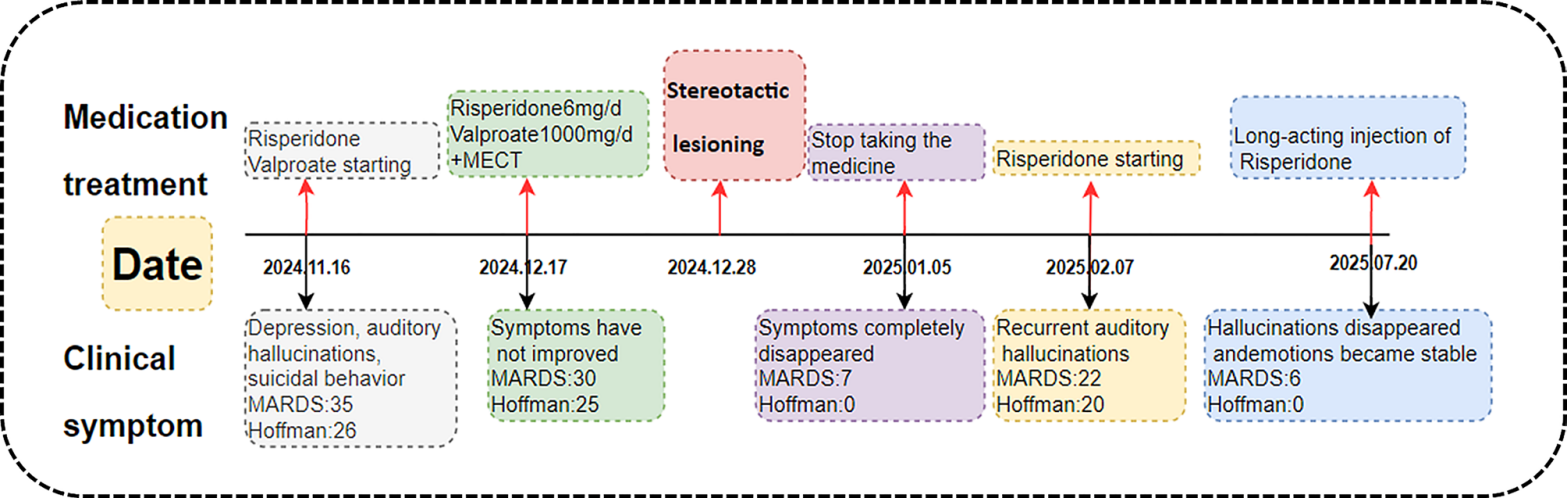
Treatment timeline of this admission

On admission in November 2024, assessments showed Montgomery-Asberg Depression Rating Scale (MADRS) [8, 9] score 35 and Hoffman Hallucination Scale [10] score 26. She received risperidone oral solution (6 ml/day), magnesium valproate (1000 mg/day) for 1 month, and ECT stopped after 12 sessions due to no meaningful response (MADRS only down from 35 to 30, persistent suicidality); seizure adequacy (45–60 s duration, PSI > 50%); optimization attempts (dose increased to 130% of threshold, anesthetic switched to etomidate) (Fig. 1).

**Fig. 2:**
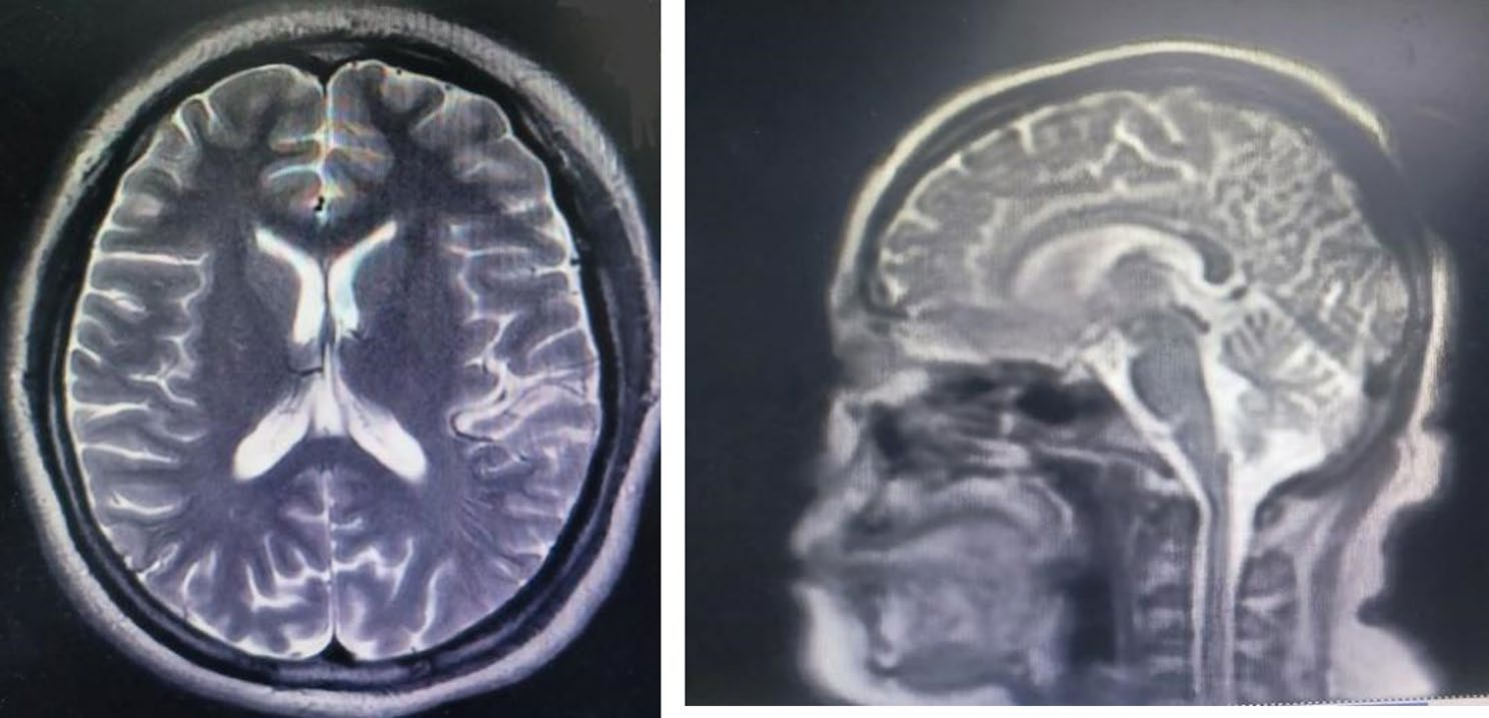
Preoperative cranial MRI

Due to refractory symptoms and family concerns about management at home, surgical intervention was requested. Following psychiatric and neurosurgical consultation, informed consent was obtained. Preoperative cranial MRI (Fig. 2) showed no abnormalities, and other preoperative tests were unremarkable.

**Fig. 3:**
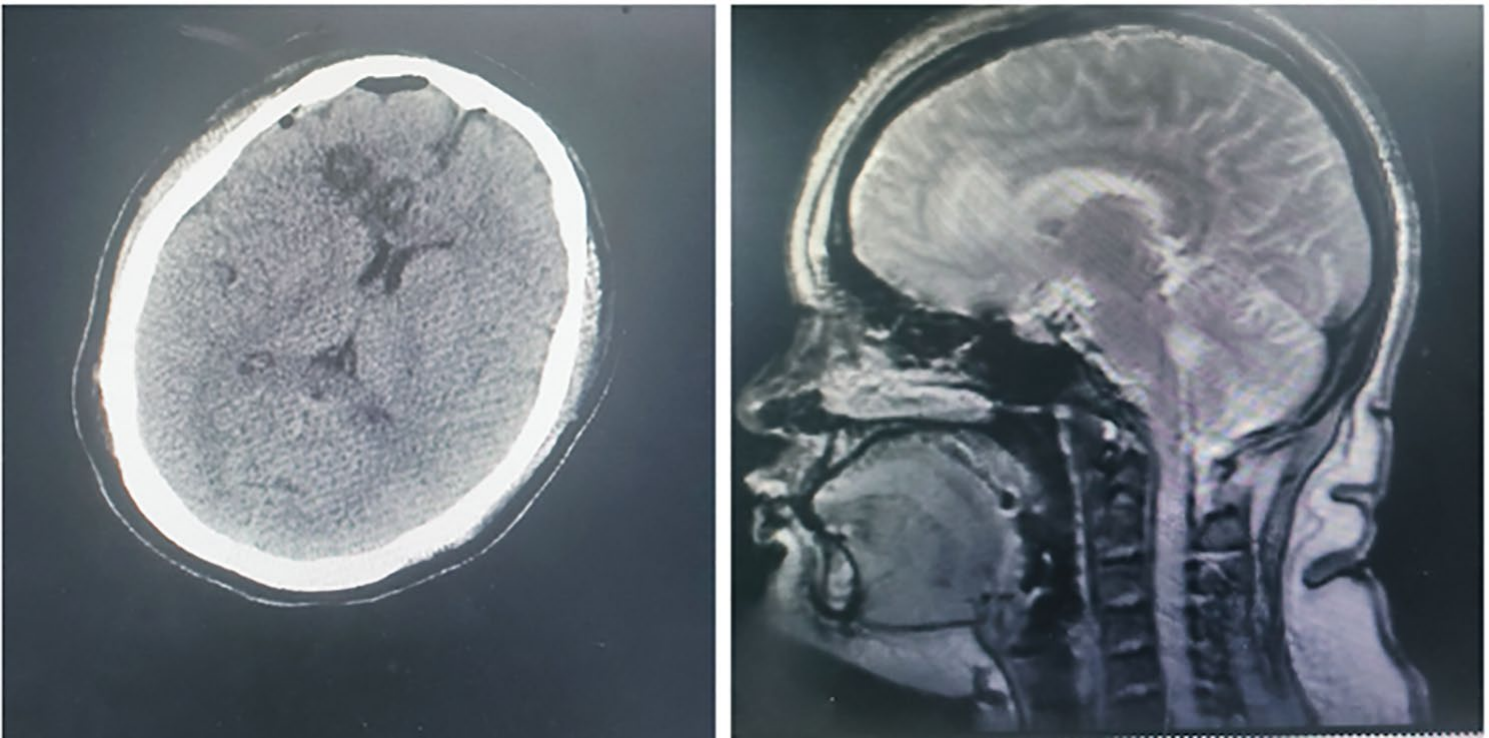
Postoperative cranial CT and MRI

SALIC-CG was performed in December 2024. The target coordinates were determined using DTI navigation: ALIC (X ± 15 mm, Y + 10 mm, Z-5 mm relative to anterior commissure-posterior commissure line) and cingulate gyrus (X ± 6 mm, Y + 20 mm, Z + 30 mm). Radiofrequency lesioning (70℃, 60s) was applied, with a lesion volume of 0.8–1.0 cm³ per side. Postoperative cranial CT and MRI (Fig. 3.) confirmed expected changes. Mild headache (NRS 3/10, resolved with acetaminophen) and transient urinary frequency (48 h resolution), no severe complications.One week postoperatively, hallucinations nearly resolved, with stable mood and no suicidal ideation/behaviors (MADRS 7, Hoffman score 0), and she was discharged. At January follow-up, auditory hallucinations recurred without self-harm (MADRS 22, Hoffman score 20); Risperidone was reintroduced, switched to long-acting injectables 1 month later. Six-month follow-up showed marked overall improvement: stable mood, absent hallucinations/negative behaviors (MADRS 6, Hoffman score 0), No cognitive decline, mild improvement at 6 months (Social function recovery (part-time work, resumed family/friend interactions).

## Discussion

The suicidal command hallucinations observed in this case of psychotic major depressive episode represent a distinct clinical entity with unique peril. Unlike command hallucinations in schizophrenia, which often lack emotional resonance, those in major depressive episode with psychotic features are tightly fused with depressive cognitions—specifically, the belief that suicide is a “deserved punishment for worthlessness” [4, 5]. This integration amplifies their coercive power, directly explaining the patient’s 10–12 daily hallucinations and 4 suicide attempts within a week. Such symptoms are not merely severe but acutely life-threatening, as evidenced by their 4.2-fold higher association with suicide compared to non-psychotic depression [2], making refractory cases (unresponsive to multiple treatments) a critical emergency requiring urgent intervention [11].

The significant alleviation of symptoms within one week following stereotactic lesioning of the anterior limb of the internal capsule (ALIC) and cingulate gyrus offers compelling evidence supporting the role of fronto-limbic circuit dysfunction in major depressive episodes with psychotic features. Neuroimaging studies have long linked the cingulate gyrus to impulsive suicidal behavior (via hyperactivity in emotional processing networks) and the ALIC to disrupted communication between prefrontal regulatory regions and limbic emotional centers [12, 13]. By disrupting this aberrant neural circuitry, the surgery likely interrupted the propagation of pathological signals that drive both psychotic symptoms (e.g., hallucinations) and suicidal urges—thereby delivering acute clinical relief. This finding aligns with the hypothesis that major depressive episodes with psychotic features involve not merely neurotransmitter imbalances, but also structural and functional abnormalities in neural networks critical to mood regulation and impulse control [14] .

A striking finding is the direct temporal relationship between antipsychotic discontinuation and symptom recurrence. The recurrence 1 month postoperatively—when glial scar formation begins to alter circuit dynamics [15]—followed by rapid stabilization upon reintroducing risperidone, confirms that surgery modulates but does not permanently normalize neural function. Risperidone, as a D2/5-HT2A antagonist, likely stabilizes residual abnormalities: its D2 blockade may dampen hyperactivity in limbic regions generating hallucinations, while 5-HT2A antagonism could restore prefrontal control over impulsive urges [16, 17]. This mirrors data showing a 76% relapse rate with postoperative antipsychotic withdrawal [18], underscoring that maintenance therapy is not an optional adjunct but a necessary component to preserve surgical benefits.

For clinical practice, this case clarifies the boundaries of surgical intervention. Stereotactic lesioning should be considered only for major depressive episode with psychotic features patients with life-threatening suicidal command hallucinations that are truly refractory: defined as failure to respond to ≥ 3 antipsychotics (from different pharmacologic classes, at maximum tolerated doses for ≥ 6 weeks) and ≥ 12 sessions of electroconvulsive therapy [4]. This strict criterion ensures surgery is reserved for cases where all conventional options have been exhausted, minimizing unnecessary risk. This pharmacotherapeutic strategy is paired with structured follow-up monitoring (monthly evaluations for the first 6 months, followed by quarterly assessments thereafter, with a minimum duration of 12 months) to enable the early detection of relapse signs.

This case also highlights the innovative potential of a “surgery + long-term pharmacotherapy” paradigm. Surgery provides acute disruption of pathological circuits to halt imminent danger, while pharmacotherapy offers chronic stabilization of residual neural activity—addressing the limitations of either approach alone (e.g., surgery’s inability to permanently normalize circuits, pharmacotherapy’s failure in acute refractory cases). For major depressive episode with psychotic features, this synergy may be particularly critical: antipsychotics not only prevent psychotic relapse but also maintain frontal-limbic balance to ward off depressive recurrence [19].

Limitations include the single-case, which restricts generalizability—factors like patient age, lesion extent, or comorbidities may influence outcomes. The patient’s young age, tailored lesion parameters, and individual characteristics restrict generalizability; clarified the case provides a preliminary roadmap rather than universal guidelines. Future research should validate this paradigm in larger cohorts, explore optimal lesion parameters (e.g., ALIC/cingulate gyrus targeting precision), and compare efficacy with less invasive neuromodulation (e.g., deep brain stimulation). Nonetheless, this report provides a critical roadmap for managing high-risk, treatment-refractory major depressive episode with psychotic features, where timely intervention can mean the difference between life and death.

## Data Availability

This article contains the key data collected in the course of this study. The datasets utilized for the present research can be obtained from the corresponding author upon reasonable request. All figures employed in the study are original works created by the authors themselves.
